# Effect of thermomechanical aging on marginal fit of three CAD-CAM restorative materials: An in vitro study

**DOI:** 10.34172/joddd.41265

**Published:** 2024-09-07

**Authors:** Reda Eid Attia, Hussein Ramadan Mohammed, Khaled Mohamed Haggag

**Affiliations:** Crown and Bridge Department, Faculty of Dental Medicine, Al Azhar University, Assiut, Egypt

**Keywords:** CAD/CAM, Hybrid ceramics, Marginal fit, Reinforced composite resin, Thermomechanical aging

## Abstract

**Background.:**

The present study assessed the impact of thermomechanical aging on the marginal fit of hybrid ceramic, reinforced composite resin, and lithium disilicate ceramic.

**Methods.:**

Eighteen human upper first premolars freshly extracted for orthodontic purposes were used to fabricate 18 CAD-CAM veneers and divided into three groups based on materials used: group H (n=6): hybrid ceramics (Vita Enamic), group R (n=6): reinforced composite resin (Brilliant crios), and group L (n=6): lithium disilicate ceramic (IPS e-max CAD). Each material’s cementation procedure was carried out according to the manufacturer’s instructions. The vertical marginal gap between the laminate veneer margin and the prepared tooth finish line was measured to assess the specimens by digital microscopy. Subsequently, all the samples were thermomechanically cycled (TMC) (5 °C to 55 °C, 30 seconds, 75000 cycles). Then, the vertical marginal gap was re-evaluated.

**Results.:**

The Brilliant crios group displayed a significantly lower vertical marginal gap mean score (31.36±2.82 µm) followed by Vita Enamic (39.27±6.54 µm) and E max (41.39±3.97 µm) groups. Similarly, after thermomechanical aging, the Brilliant crios group displayed a significantly lower vertical marginal gap mean score (41.83±8.28 µm) followed by Vita Enamic (55.47±18.65 µm), and the E max group showed the greatest vertical marginal gap mean score (59.43±16.27 µm).

**Conclusion.:**

Thermomechanical aging affected the marginal fit of different materials employed in the current research, and these changes were under the established clinical limit.

## Introduction

 Laminate veneers are indirect restorations that restore adequate shape and function and harmonize the smile and color of aesthetically compromised teeth.^1^ The material of choice for laminate veneers is dental ceramic because of its esthetic advantages, including improved fluorescence, increased translucency,^2^ and high capacity to mimic tooth structure. In addition, dental ceramics are inert, biocompatible, and resistant to corrosion and have low electrical and temperature conductivity.^3,4^

 In addition to the aesthetics and mechanical strength of any restoration,^5^ marginal fit is an essential requirement that must be carefully considered in the final restoration.^6^ Possible spaces might form across the prepared tooth and the external restoration boundary due to an inadequate fit. As these spaces increase, and as the majority of luting cement dissolves in oral fluids, bacterial plaque may accumulate in such compromised regions, causing pulpal lesions, caries, and gingival irritation. Additionally, disturbances in the fit can create stress concentrations and weaken the restoration, ultimately leading to its fracture.^7^

 The globe’s initial hybrid dental ceramic with a double network design is called Vita Enamic. The acrylate polymer network in this dental material reinforces the predominate fine-structure ceramic network (86% by weight), and both networks are completely interwoven.^8^

 Creating resin matrices for CAD-CAM blocks is a crucial step in optimizing CAD-CAM substances. Novel materials known as hybrids may be further classified into two categories depending on their chemical formulations: materials with a predominance of ceramic elements, known as resin nanoceramics, and materials primarily composed of resin matrix, known as nanohybrid composite resins. Brilliant crios (BC) is a novel CAD-CAM material that is a nanohybrid composite resin block. Backed by a strongly cross-linked methacrylate matrix, BC’s composition comprises around 71 wt% of inorganic filler that grasps 20-μm silica and 1-μm barium glass, leading to superior marginal performance over glass ceramics and increased dentin adhesion.^9^

 Since BC’s modulus of elasticity is similar to that of dentin, which is significantly lower than that of ceramics, it creates a unique biomechanical compound (monoblock), where the restoration and tooth work together to more uniformly distribute force and increase the flexural strength.^9^ Furthermore, the reinforced composite has outstanding mechanical qualities thanks to controlled manufacturing and continuous heat curing, which might make them ideal for use in locations with strong occlusal stresses.^10^

 Lithium disilicate ceramic is one of the ceramic systems used to fabricate monolithic restorations. Due to its better physical qualities and aesthetics, it has been successful over a long period. It is, therefore, more widely accepted for the restoration of the anterior and posterior teeth.^11,12^

 There are variations as to the extent to which the marginal fit is considered clinically acceptable. McLean and von Fraunhofer^13^ proposed that in clinical terms, the restoration’s margin is deemed acceptable if the cement thickness and marginal gaps are < 120 µm.

 Fransson et al^14^ showed that following cementation, a marginal gap of < 150 µm ought to be considered clinically acceptable. Another research proposed that it might be deemed acceptable whenever the marginal fit is invisible to the human eye or cannot be found using a dental probe.^15,16^ Moreover, Mclean and von Fraunhofer^13^ evaluated the marginal fit of 1000 fixed restorations for five years, concluding that it is challenging to identify a marginal gap measuring < 80 μm in a clinical setting.

 There are four basic techniques for marginal fit measurement: direct view, cross-sectional view, impression replica method, and explorer and visual inspection.^17^

 It is important to evaluate how different kinds of veneer materials behave in the oral cavity to fabricate durable veneers for patients. Variables in the oral cavity, including temperature, pH, humidity, and the veneer material’s resistance to such alterations, impact their durability over time.^18,19^ To assess the restorative materials’ capacity to remain unaltered, artificially accelerated aging is used to replicate the oral environmental circumstances.^20^

 The current in vitro study assessed the impact of thermomechanical aging on the marginal fitof ceramic veneers made from VITA Enamic, Brilliant Crios, and IPS e-max CAD.

## Methods

Table 1 lists the materials used in this study.Based on a previous study,18 upper first premolars recently extracted for orthodontic purposes were chosen.^21^ The premolars had no decay, microfractures, or abrasion cavitiesand did not have endodontic treatment in the past with a somewhat comparable size and form. The chosen premolars were divided equally into three groups according to the type of ceramic materials used to construct laminate veneers.

**Table 1:** The materials used in this study

**Materials**	**Composition**	**Manufacturer**	**LotNo**
Dental hybrid ceramic Vita Enamic	-Ceramic part 86 % (SiO_2_, Al2O3, Na_2_O, K_2_O, ZrO_2_) -Polymer part 14 % (UDMA, TEGDMA)	VITA Zahnfabrik, Germany	61960
Nano ceramic reinforced resin composite Brilliant crios	Barium glass < 1.0 µm, Amorphous silica SiO < 20 nm Resin matrix: Cross-linked methacrylates Pigments: ferrous oxide and titanium dioxide.	Coltene/Whaledent AG, Switzerland	L 33922
Lithium Disilicate IPS e-max CAD	SiO_2_ Additional contents (Al_2_O_3_, K_2_O, CaO, Na_2_O, other oxides)	Ivoclar Vivadent AG, Schaan/ Liechtenstein Germany	Z01SBK

 A custom-made Teflon mold was machine-milled and used to fabricate resin blocks. Each premolar was mounted separately using a self-cured acrylic resin (Acrostone Dental Factory, England.) vertically along its long axis. A hollow cylinder with an internal diameter of 2 cm and a depth of 2 cm was placed in the mold. The mold was held on the base of the paralleling device (Paraflex, Bego, Bremer, Germany) by screws. Each premolar was fixed to the lower tip of the device via a sticky wax; then, the tip was moved down to the predetermined depth, where the cervical line was 1 mm away from the self-cured acrylic resin, and waited for the final setting. The putty consistency of additional silicon impression material (Via L. Longo, 18-50019, Sesto F.no, Firenze, Italy) was used to create indexes for each tooth, ensuring uniform tooth preparation.

 The natural tooth was prepared using a standardized preparation bur set (6926 Via al Molino 107, Switzerland) for ceramic laminate veneer fabrication. Occlusal reduction was approximately 1.5-mm^22^ clearance, and preparation went 2 mm toward the central groove. The buccal reduction was 0.5 mm. The proximal reduction was positioned immediately after the mediolabial and distolabial line angles. The cervical margin was positioned 1 mm^23^ occlusal to the cementoenamel junction. The preparation margin was surrounded by a chamfer finish line; its continuity and consistency were examined, and a rubber index was used to assess the degree of tooth reduction.

 All restorations were fabricated by CAD-CAM system (Imes-Icore CAD-CAM System. Germany), which consists of a personal computer, a DOF scanner (Degree of Freedom Scanner, Korean), and an Imes-Icore milling unit. The in-lab user interface window was used to enter the restoration information. Veneer was chosen as the restoration type, and the upper first premolar was selected as the tooth and design mode. In the next step, the operator started the scanning process using Telescan spray (DFS-Diamon GmbH-Germany) to facilitate the acquisition of optical impressions for the prepared teeth. Scanning was carried out by a movable camera with a 3-axis scanning arm, which made it easier and quicker.

 The laminate veneers were designed by the Exocad system (Exocad GmbH, Julius-Reiber-Str. 37, D-64293 Darmstadt), and restoration parameters were set, including spacer thickness (50 µm), the minimum thickness of the material. The model tooth veneer was selected, loaded, and placed on the virtual die image. The milling procedure was initiated by activating the milling preview window via a 5-axis dental milling machine. The milling procedure was accomplished with an abundant spray of water. Following the milling procedure, a low-speed diamond bur (Komit, Gebr, Brassrler, Lemgo, Germany) was used to eliminate excess material at the point of junction with the ceramic block and divide the fully milled restorations.

 Based on the manufacturer’s directions, polishing, and finishing procedures were applied to Vita Enamic CAD/CAM restorations using a two-stage Vita Enamic polishing kit (Vita Zahnfabric, Bad Sackingen, Germany). The restorations made by Brilliant crios were subjected to finishing and polishing procedures using a Diatech finishing and polishing kit. (Coltene/Whaledent AG, Switzerland) The programat P310 oven (Ivoclar Vivadent AG Bendererstr. 29494 Schwan Liechtenstein, Germany) was used for the crystallization of IPS e.max CAD specimens and fired on their special firing tray based on the manufacturer’s instructions.

 The inner surfaces of the Vita Enamic specimens were etched with 5% hydrofluoric acid (HF) for 60 seconds (Bisco, Inc. 1100 W, Schaumburg, IL 60193 U.S. A), rinsed, and air-dried based on the supplier recommendations and placed in an ultrasonic cleaner (Vita Sonic II, Germany) with distilled water for 5 minutes to remove the acid and debris remaining after acid etching. A silane coupling agent was applied for 60 seconds (Bisco, Inc. Schaumburg, USA) and left to dry.

 The inner surfaces of the Brilliant crios specimens were sandblasted with 40-μm aluminum oxide particles at 1.5 bar for 15 seconds from a distance of 10 mm perpendicular to the inner surface of the specimen using the sandblasting device and bonded with a special bonding agent (One Coat 7 Universal) for Brilliant crios (Coltene Ltd – 60019539, Switzerland).

 The inner surfaces of the E-max specimens were acid-etched with 9.5% HF for 20 seconds, followed by a 60-second water washing and placing in an ultrasonic cleaner for 5 minutes to remove the acid and debris remaining after acid etching. A silane coupling agent was applied to the inner surface for 60 seconds.

 The enamel surface of all the teeth used in this study was etched with 37% phosphoric acid gel for 30 seconds (Madespa S.A Calle Río. Jarama, 12045007 Toledo), washed for 15 seconds with plenty of water, and air-dried. A universal adhesive (All Bond Universal Adhesive, Bisco, Inc.1100 Schaumburg, IL, 60193, USA) was applied to the etched enamel and allowed 15 seconds for reaction followed by 5 seconds of air drying without light curing according to manufacturer’s instructions.

 The light-cured resin cement Choice 2 (Bisco, Inc., Schaumburg, USA) was applied to the produced veneer bonding surface. A specifically made cementing tool was used to provide a constant stress of 1 kg for 20 seconds^24^ on the buccal surface of the veneers that had been cemented.

 The excess cement was light-cured for 2 seconds, eliminated with an explorer while the cement was still in its gel condition, and each veneer surface was light-cured again for 15 seconds at 1400 mW/cm^2^ (Monitex, LiteQ LD-107, Cordless LED Curing Light, Taiwan). Each tooth was again immersed in 37 ºC distilled water after restorative bonding until assessment.

###  Marginal fit evaluation before thermomechanical aging

 The specimens (n = 6 in each group) were captured on video using a USB digital microscope with an integrated camera (U500X Digital Microscope, Guangdong, China) fixedly magnified by × 40 and linked to a suitable laptop.

 The gap size was determined and assessed via a computerized image analysis program (Image J 1.43U, National Institute of Health, USA). All boundaries, dimensions, frames, and assessed parameters within the ImageJ program were given in pixels. Consequently, system calibration was carried out to translate the pixels into exact real-world units. A ruler (a recognized element utilized in the current study) was compared to a scale produced by the ImageJ program for calibration. The specimens were kept in position with a specially created holding mechanism.

 For every specimen, shots of the margins were obtained. After that, morphometric measurements were made for every picture using three marks spaced equally throughout each surface’s perimeter. Every point was then measured three times. The acquired data were gathered, tabulated, and then subjected to statistical analyses.

###  Thermomechanical cyclic loading

 For thermal cyclic aging, 7500 cycles were employed. Every water bath had a dwell time of 25 seconds and a lag time of 10 seconds. The minimum temperature level was 5ºC while the maximum temperature level was 55 ºC. A computerized logic-controlled apparatus was used for mechanical aging; a servomotor was used to run the recently created four-station multimodal ROBOTA chewing simulator combined via a thermocycling process. In the bottom sample holder, the samples were implanted in Teflon casing. Five kilograms of weight was used, corresponding to 49 N of chewing force. To simulate six months of chewing situation clinically, the examination was performed 75 000 times.

###  Marginal fit evaluation after thermomechanical aging

 After the thermomechanical aging of specimens, the vertical marginal gap was measured using the same technique as before thermomechanical aging.

###  Statistical analysis

 Means and standard deviations were used to describe the data, following confirmation of the normal distribution of errors and the uniformity of variance. Post hoc Tukey tests were used when the results of one-way ANOVA indicated statistical significance. The significance level was set at *P* ≤ 0.05.

## Results

**Figure 1:**
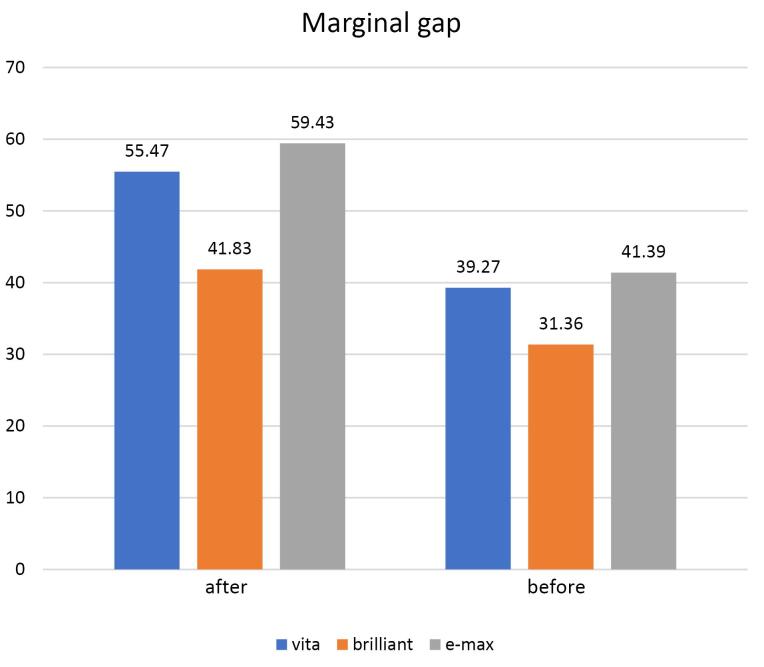
Vertical marginal gap (μm) values for all groups before and after thermomechanical aging

 Vertical marginal gap (μm) results as mean and standard deviation (SD) values for all groups before and after thermomechanical aging are summarized in Table 2 and graphically drawn in Figure 1.

**Table 2:** Vertical marginal gap results (Mean values ± SDs) for all the groups before and after thermomechanical aging

**Variable**		**Thermomechanical aging**								**Statistics**
		**Before**				**After**				
		**Mean**	**±SD**	**95% CI**		**Mean**	**±SD**	**95% CI**		**t-test**
				**Low**	**High**			**Low**	**High**	***P*** ** value**
Material group	Vita Enamic	39.27^A^	6.54	35.57	42.97	55.47^A^	18.65	63.3	70.32	< 0.0001*
	Brilliant crios	31.36^B^	2.82	29.77	32.96	41.83^B^	8.28	37.14	46.51	0.004*
	E.max CAD	41.39^A^	3.97	39.15	43.64	59.43^A^	16.27	51.92	84.4	0.0075*
Statistics	ANOVA	*P* value	0.0003*			*P* value	0.0008*			

* Statistically significant. Values with same superscript letter are not significantly different.

 Vita Enamic group recorded a significantly higher vertical marginal gap mean value after aging (55.47 μm) than before (39.27 μm) based on paired t-test (*P* ≤ 0.000). The Brilliant crios group exhibited significantly higher vertical marginal gap mean value after aging (41.83 ± 8.28 µm) than before aging (31.36 ± 2.82 µm) based on paired t-test (*P* = 0.0075). The e.max CAD group had a significantly higher vertical marginal gap mean value after aging (59.43 ± 16.27 µm) than before (41.39 ± 3.97 µm) based on paired t-test (*P* = 0.004).

###  Total effect of material group on marginal fit

 Regardless of aging, the differences between groups were statistically significant according to two-way ANOVA (*P* = 0.0001) (e.max CAD ≥ Vita Enamic > Brilliant crios).

###  Aging’s impact on the marginal fit

 Regardless of material groups, all the groups had a significantly higher mean value for the vertical marginal gap after aging than before, according to the two-way ANOVA (*P* ≤ 0.0001).

Table 2 and Figure 1 show vertical marginal gap results (mean ± SD) for all the groups before and after thermomechanical aging.

## Discussion

 Due to enhanced mechanical and optical properties, glass-matrix ceramics are frequently used for CAD/CAM restorations. Although they are well-established and successful materials, they have several disadvantages; due to hardness, glass-matrix ceramics have mechanical problems such as brittleness and abrasion on the opposing dentition.^25^

 New restorative materials called polymer-infiltrated ceramics and composite CAD for use with CAD/CAM systems have been developed to improve the unfavorable properties of glass-matrix ceramics (high brittleness index and the need for further heat treatments after milling).^26^

 The advantage of polymer-based materials is their good machinability, removing the need to fire restorations after milling. Also, they have a low brittleness index (which substantially reduces marginal chipping during the manufacturing process) and have elastic moduli comparable to those of natural tooth substances.^27,28^

 Within the present research, every sample’s marginal fit was assessed at twelve different positions, three of which were at each of the four margins: the buccal, mesial, distal, and occlusal.^29^ The average value across all measurements was determined. Each evaluation was carried out by the same operator to minimize statistical variation.

 The marginal fit was assessed using a digital microscope with a fixed magnification of × 40 for direct inspection and exterior evaluations.^30^ Because of its noninvasive nature, this measuring method helps determine the exact fit of the entire sample margin.

 Extracted natural teeth were used as samples since they stimulate clinical situations more closely than resin abutments. For standardization purposes to decrease the differences between natural teeth and make the preparation more identical for all specimens, only maxillary first premolars with average dimensions were used in this study.^31^

 Every single tooth was included in the silicone index before reduction for standardization. To prevent variations in preparation dimensions, standardized tooth preparation was carried out.^32^ For the optimal marginal fit, the recommended finish line types include shoulder-bevel, chamfer, and shoulder. Prior research revealed no distinction between the shoulder’s marginal fit and the chamfer margins; nonetheless, the chamfer design offers several clinical benefits over the shoulder design.^33^ For the best adhesion of the veneers to the tooth structure, the preparation must occur in the enamel; hence, 0.5–0.7 mm of enamel preparation is advised before applying veneers.^34^

 The recently created four-station chewing simulator, powered by a servomotor and combined with a thermocycling protocol, carried out the cyclic loading procedure.^35^ A vertical load of 50 N was applied, which is thought to represent the mean of the physiological forces generated by mastication in non-bruxism patients’ teeth.^36^ A total of 75 000 cycles were completed, simulating six months of regular activity.^35^

 Before thermomechanical aging, the Brilliant crios group showed a significantly lower vertical marginal gap average score (31.36 ± 2.82 µm) followed by Vita Enamic (39.27 ± 6.54 µm) and e-max group (41.39 ± 3.97 µm). Similarly, after thermomechanical aging, the Brilliant crios group showed a significantly lower vertical marginal gap average score (41.83 ± 8.28 µm), followed by Vita Enamic (55.47 ± 18.65 µm), and the e-max group showed the highest vertical marginal gap average score (59.43 ± 16.27 µm). These results rejected the null hypothesis.

 Our research findings agreed with the investigation that assessed the marginal gaps between laminate veneers made using Lava Ultimate, IPS e-max CAD, and IPS Empress CAD on an epoxy resin die that had been produced and sealed using resin cement. All the samples’ marginal gaps were determined and documented before and after the artificially accelerated aging procedure. Both material type and aging process had a statistically significant impact on the marginal gap, which might be attributed to variations in the materials’ constitution. Restoration precision may be impacted by filler particle size and the kind of milling bur used. Additional factors include the CAD-CAM system’s inherent characteristics, the milling device’s rotary tool selection and speed, the production process, the preparation design, the spacer’s thickness, the precision of the scanning technique, the program system, and the kind of restorative material.^37^

 Furthermore, compared to lithium disilicate ceramics, Brilliant crios materials are likely less brittle, contributing to this outcome. Because of its greater hardness, milling lithium disilicate requires more effort and time, which might raise the marginal disparity.^38^ Additionally, Azarbal et al^39^ have shown that the hardness of lithium disilicate may contribute to the wear of CAD/CAM milling burs because repetitive milling may impair the burs’ capability to cut, leading to increased marginal differences.

 In the present study, thermomechanical aging caused a significant rise in the mean scores of the marginal gaps, which can be explained by the accelerated hydrolysis of unprotected collagen fibers and extraction of improper polymerized resin tags due to exposure to hot water. In addition, generated stresses at the tooth‒restoration interface because of a mismatch in the coefficient of thermal expansion of tooth structure and restorative material have been suggested as a crucial factor for deterioration of the marginal adaptability.^40^

 These temperature alterations can cause expansion and contraction of the restorative materials, resulting in stresses, crack formation, and propagation due to an imbalance between the filler particles and the resin matrix’s coefficients of thermal expansion.^41^

 Yao et al^42^ reported that the marginal accuracy of the CAD/CAM interim composite crowns did not change. Regarding the ideal marginal gap for ceramic crowns, a few studies have reported that the ideal marginal gap should be 25‒40 μm for cemented restorations.^43^ Other studies considered the marginal gap values of 100‒200 μm clinically acceptable for cemented restorations.^44,45^ More recent studies have evaluated the clinically good deals of the marginal gap to be less than 100 µm.^30^ Therefore, the results of marginal gaps for all groups presented in this study can be considered clinically acceptable.

 One of the present study’s limitationswas thatthermomechanical aging was performed for a limited number of 7500 thermal cycles corresponding to only six months of clinical use, so more research is needed to simulate long-term oral performance for better evaluation of the durability of veneer restorations. Also, artificial saliva should be incorporated.

## Conclusion

 The subsequent clinical suggestion and conclusion might be made within the constraints of the present research:

- Every restorative material that has been evaluated was susceptible to vertical marginal gap changes after being subjected to thermomechanical aging.
- Brilliant crios material exhibited the least marginal gap before and after thermomechanical aging.
- The vertical marginal gap of every material evaluated before and following thermomechanical loading was within the clinically acceptable limit.

## Competing Interests

 No competing interests.

## Ethical Approval

 This study was approved by the Research Ethics Committee Faculty of Dental Medicine, Al-Azhar University, under protocol number EC. Ref No:644/3634.
